# Burden of Diabetes Mellitus Estimated with a Longitudinal Population-Based Study Using Administrative Databases

**DOI:** 10.1371/journal.pone.0113741

**Published:** 2014-12-03

**Authors:** Luciana Scalone, Giancarlo Cesana, Gianluca Furneri, Roberta Ciampichini, Paolo Beck-Peccoz, Virginio Chiodini, Silvia Mangioni, Emanuela Orsi, Carla Fornari, Lorenzo Giovanni Mantovani

**Affiliations:** 1 CESP – Research Centre on Public Health, University of Milano Bicocca, Milan, Italy; 2 CHARTA Foundation, Milan, Italy; 3 Unit of Endocrinology and Diabetology, Department of Medical Sciences, Fondazione IRCCS Cà Granda - Ospedale Maggiore Policlinico, University of Milan, Milan, Italy; 4 Endocrinology and Diabetes Unit, Department of Medical Sciences, Fondazione IRCSS Cà Granda-Ospedale Maggiore Policlinico, San Giuseppe Hospital, Milan, Italy; Medical College of Soochow University, China

## Abstract

**Objective:**

To assess the epidemiologic and economic burden of diabetes mellitus (DM) from a longitudinal population-based study.

**Research Design and Methods:**

Lombardy Region includes 9.9 million individuals. Its DM population was identified through a data warehouse (DENALI), which matches with a probabilistic linkage demographic, clinical and economic data of different Healthcare Administrative databases. All individuals, who, during the year 2000 had an hospital discharge with a IDC-9 CM code 250.XX, and/or two consecutive prescriptions of drugs for diabetes (ATC code A10XXXX) within one year, and/or an exemption from co-payment healthcare costs specific for DM, were selected and followed up to 9 years. We calculated prevalence, mortality and healthcare costs (hospitalizations, drugs and outpatient examinations/visits) from the National Health Service’s perspective.

**Results:**

We identified 312,223 eligible subjects. The study population (51% male) had a mean age of 66 (from 0.03 to 105.12) years at the index date. Prevalence ranged from 0.4% among subjects aged ≤45 years to 10.1% among those >85 years old. Overall 43.4 deaths per 1,000 patients per year were estimated, significantly (p<0.001) higher in men than women. Overall, 3,315€/patient-year were spent on average: hospitalizations were the cost driver (54.2% of total cost). Drugs contributed to 31.5%, outpatient claims represented 14.3% of total costs. Thirty-five percent of hospital costs were attributable to cerebro−/cardiovascular reasons, 6% to other complications of DM, and 4% to DM as a main diagnosis. Cardiovascular drugs contributed to 33.5% of total drug costs, 21.8% was attributable to class A (16.7% to class A10) and 4.3% to class B (2.4% to class B01) drugs.

**Conclusions:**

Merging different administrative databases can provide with many data from large populations observed for long time periods. DENALI shows to be an efficient instrument to obtain accurate estimates of burden of diseases such as diabetes mellitus.

## Introduction

Diabetes Mellitus is a common chronic disease and exerts a heavy burden on society of our time because of its increasing prevalence, the chronic nature of disease and the high risk of major complications: blindness, renal disease, foot ulcers and cardiovascular disease which is the major cause of death, accounting in most populations for 50% or more of all diabetes fatalities and much disability [1]. The world prevalence of diabetes was estimated to be 6,4% in 2010 and to rise to 7,7% in 2030 [2]: these projections are somewhat higher than predictions made only a few years ago [3]; type 2 diabetes is the predominant form and accounts for at least 90% of cases [4]. According to the estimate of the Italian national institute of statistics [5] in 2010, in Italy there were over3 million people with type 2 diabetes diagnosed and another 1 million people with the same disease undiagnosed. The increasing prevalence of diabetes is dependent by the demographic changes, including improved health status, ageing of populations, progressive urbanization, and also by the worse lifestyle, including high-energy diets and reduced physical activity [6]–[8]. Diabetes prevalence along with management of its complications represent a key driver behind total diabetes direct costs: the London School of Economics processed recently an analysis [9] about the trend of the diabetic disease in the main five European countries(Germany, France, Italy, UK, Spain) and found that inpatient costs were consistently higher than outpatient costs in all countries due to increased medical care required with diabetes related complications. In Italy total direct costs amounted to 8€ billion in 2010 [10] with a cost rate ratio between diabetic and not-diabetic patients equals to 1.54 [11]. Tracking and monitoring chronic diseases such as diabetes is essential in public health surveillance for defining the burden of disease, for planning health services, for evaluating strategies in prevention, control and outcome assessment. Administrative databases can constitute useful and efficient instrument for that objective, in particular if they can provide with accurate data from a large amount of individuals observed for conspicuous time periods. Objective of the present study was to estimate prevalence, healthcare costs, occurrence of complications and mortality of diabetes mellitus with a longitudinal population-based study through the combination of several healthcare administrative databases.

## Research Design and Methods

### Data source

Data were extracted from healthcare administrative databases of Lombardy, a northern Italian region with a population of about 9.9 million members (16.4% of the Italian population). The available administrative datasets regarded: hospital discharges, pharmaceutical prescriptions, outpatient claims (laboratory and diagnostic examinations, specialist medical visits) and related costs covered by the Regional Health System (RHS), which pays for most of them. RHS is funded by the National Health System and provide a “universal coverage” to all its residents, who generally have to pay only a part of costs of drugs or services (ticket). In case people have a specific condition, such as a severe chronic disease like diabetes mellitus, they are exempted from co-paying. The exemption is allowed through the assignment of an exemption code, which we used as a mode to identify diabetes patients.

These datasets were merged with others containing demographic characteristics (gender, place and date of birth, residence) and with the disease-specific exemption registry, which includes identification of subjects exempted from co-paying drugs and services due to their chronic disease condition. The databases were combined using a data warehouse named DENALI, to facilitate extraction, processing and analysis. One of the distinguishing features of DENALI is the probabilistic reconstruction of links (probabilistic record linkage) [12], to match data of the different datasets belonging to the same individuals.

### Study Population

The study population consisted of all subjects who during the year 2000 (from January 1^st^ to December 31^st^) met at least one of the following criteria: hospital discharge with a ICD-9 CM [International Classification of Diseases] code 250.XX in at least one of the six discharge diagnosis fields; two consecutive prescriptions of drugs for diabetes within one year of observation, classified with the Anatomical Therapeutic Chemical (ATC) classification system [code A10XXXX]; an exemption from co-payment healthcare costs specific for diabetes mellitus (code 13.250).

The index date was defined as the date of occurrence of the first criterion above. Only data of patients with a valid status (i.e., not dead or migrated to other places) in the regional archive at the index date were included in the analyses. The individuals eligible for the analyses were followed until 31^st^ December 2008 or until death or emigration.

### Data and analyses

The characteristics of the study population were described with absolute and relative (percentages) numbers or as minimum (min), maximum (max), median, mean and standard deviation (±SD). Prevalence of diabetes was calculated dividing the number of subjects meeting the eligibility criteria at the index date by the number of Lombardy residents at January first 2001 [13], stratified by sex and age classes.

General mortality rates, also stratified by sex and age were estimated, applying a survival function over nine years with the nonparametric Kaplan-Meier method. The equality of survival functions between gender and age classes was tested using the log-rank test. Hence, mortality rates were calculated dividing the number of deaths by the person-time until death obtained with the survival function. Mortality rates were reported along with their 95% CI, calculated using the quadratic approximation to the Poisson log likelihood for the log-rate parameter. Mortality rate comparison between genders was performed using the Mantel-Haenszel test for unequal rate ratios (RRs). Direct standardization method was adopted to estimate comparable mortality rate using the 2001 Italian population as standard [13].

Healthcare costs were analyzed from the perspective of the RHS. Costs were computed using charges that the RHS reimbursed to the providers of care. Three main cost categories were identified: hospitalizations, pharmaceutical prescriptions and outpatient claims.

Hospitalizations were stratified into four groups, according to the following main diagnosis, of the Diagnosis Related Group (DRG) code and related tariff: group 1, diabetes (ICD-9 codes: 250.X), group 2, cardio- or cerebro-vascular events (ICD-9 codes: 40X.X; 41X.X; 42X.X; 43X.X; 44X.X; 451.X; 452.X; 453.X), group 3, other possible DM related complications (ICD-9 codes: 337.X, 354.X, 355.X, 357.X, 358.X, 362.X, 365.X, 366.X, 369.X, 459.X, 581.X-589.X, 707.X, 713.X, 785.X, 895.X-897.X), group 4, any other main diagnosis. Pharmaceutical claims were grouped according to the ATC system to distinguish costs related to drugs for alimentary tract and metabolism (A), drugs for blood and blood forming organs (B) and drugs for cardiovascular system (C). We further grouped pharmaceutical claims to focus on insulin (A10A), other blood glucose lowering drugs (A10B) and antithrombotic agents (B01A). Finally, we estimated outpatient costs using outpatient claims for instrumental examinations, laboratory tests and specialist medical visits.

For analysis of costs, means were used as central tendency parameter, expressed as mean cost (€) per patient per year. Hospital admissions were also presented as frequency of utilization according to the main diagnosis. Because of the highly skewed distribution of cost variables, we report, as a variability measure, the distribution of costs per patient per year, instead of standard deviations.

All analyses were performed using STATA version 11.0 (StataCorp LP, USA).

## Results

### Study population

A total of 312,223 eligible subjects were identified in the year 2000∶64.3% were identified through the co-payment exemption code, 28.2% had two consecutive diabetes drug prescriptions, 7.6% were included because of the hospital discharge with a diagnosis of diabetes mellitus. The study population was aged, at the index date, 66 years on average and consisted of 51% males. It was observed on average (±SD) for 7.29 (±2.67) years, from a minimum of 1 day (175 subjects) to a maximum of 9 years (141,476 subjects, almost half of the total study population), for a total of 2,274,645.5 patient-years (Table 1).

**Table 1:** Description of study population.

Descriptive variables	Male	Female	Total
Patients – N. (%)	153,903 (49.3)	158,320 (50.7)	312,223 (100.0)
Age classes$, years – N (%)			
< = 45	9,478 (6.2)	11,235 (7.1)	20,713 (6.6)
46–55	12,777 (8.3)	22,781 (14.4)	35,558 (11.4)
56–65	32,423 (21.1)	47,734 (30.2)	80,157 (25.7)
66–75	48,696 (31.6)	51,085 (32.3)	99,781 (32.0)
76–85	36,877 (24.0)	21,413 (13.5)	58,290 (18.7)
> = 85	13,652 (8.9)	4,072 (2.6)	17,724 (5.7)
Age$, years			
min	0.03	0.15	0.03
max	105.12	103.9	105.12
median	69.79	64.55	66.95
mean (±SD)	68.09 (±13.75)	63.4 (±12.49)	65.71 (±13.33)
Prevalence (%) by age classes			
< = 45	0.4	0.5	0.4
46–55	2.1	3.9	3
56–65	5.7	8.9	7.2
66–75	9.7	12.9	11.1
76–85	11.9	12.8	12.2
> = 86	10.3	9.5	10.1
All ages	3.3	3.6	3.5
Mortality Rate x 1,000 (95% IC)			
< = 45	2.6 (2.2–2.9)	4.8 (4.4–5.3)	3.8 (3.5–4.1)
46–55	8.41(7.9–9.0)	12.2 (11.7–12.7)	10.8 (10.4–11.2)
56–65	15.1 (14.6–15.5)	24.3 (23.8–24.8)	20.5 (20.1–20.8)
66–75	35.0 (34.4–35.6)	53.3 (52.5–54.0)	44.0 (43.5–44.5)
76–85	85.6 (84.4–87.0)	114.6 (112.7–116.6)	95.6 (94.5–96.6)
> = 86	203.9 (200.2–207.6)	250.0 (242.0–258.3)	213.3 (209.9–216.7)
All ages	44.4 (44.1–44.8)	42.1 (41.8–42.5)	43.3 (43.0–43.5)

$ Age is referred at the index date.

### Prevalence

The study population identified in our study indicated a diabetes prevalence of 3.5% in 2000, ranging from 0.4% among subjects 45 years old or younger, to 10% among those aged more than 85 years (Table 1).

### Mortality

During the observational period a total of 98,422 deaths (31.5% of study population) occurred, with an overall mortality rate of 43.3 deaths per 1,000 patients per year (Table 1). The Kaplan-Meier survival functions estimates showed that women had statistically significant (p<0.001) lower mortality rates than men in every age class, although the difference decreased with the increasing of age (from RR = 1.9 among subjects aged < = 45 to 1.2 among subjects aged> = 85 years). Controlling for age classes, the Mantel-Haenszel test for inequality mortality rate confirmed a significant (p<0.001) disadvantage in the overall mortality rate for men with respect to women. Using the direct standardization method the overall mortality rate was 17.5 deaths per 1,000 patients per year for females and 24.9 for males.

### Healthcare costs

**Figure 1:**
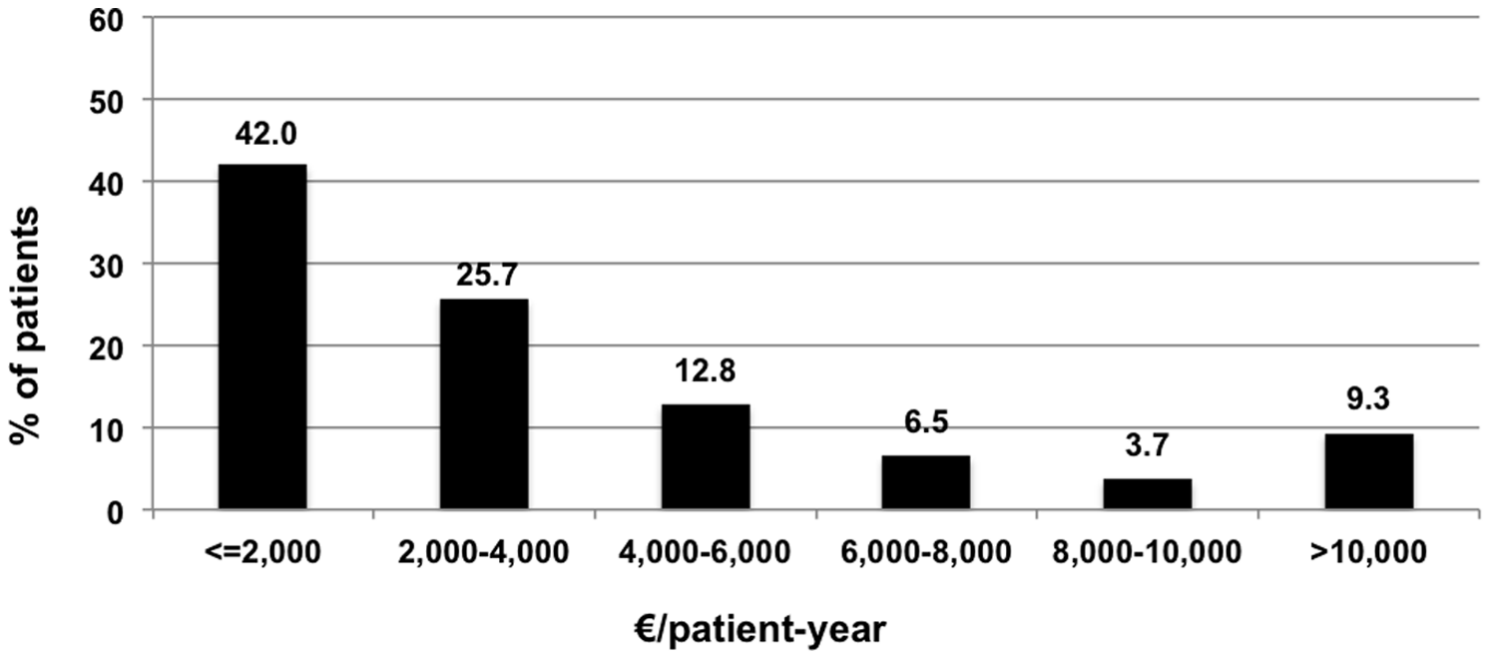
Patient distribution of mean healthcare costs (€/patient-year).

Overall, 3,315.06 euros per patient-year were spent on average. Actually, 42% of subjects cost less than 2,000 euro per patient-year, while almost 40% cost between 2,000 and 6,000 euro, 10% cost between 6,000 and 10,000 euro per patient-year, and 9% cost more than 10,000 euro per patient-year (Figure 1).

**Figure 2:**
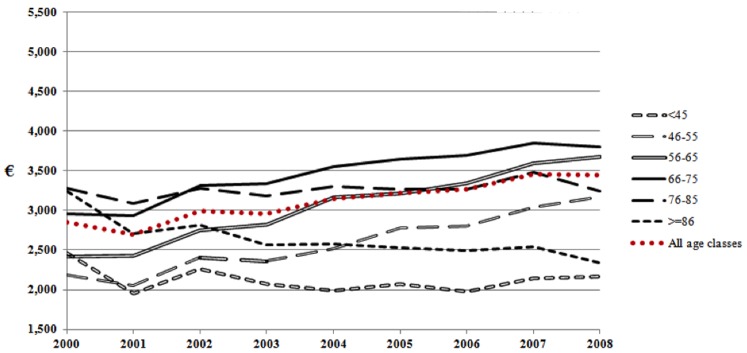
Women expenditure (€/patient-year) by age classes.

**Figure 3:**
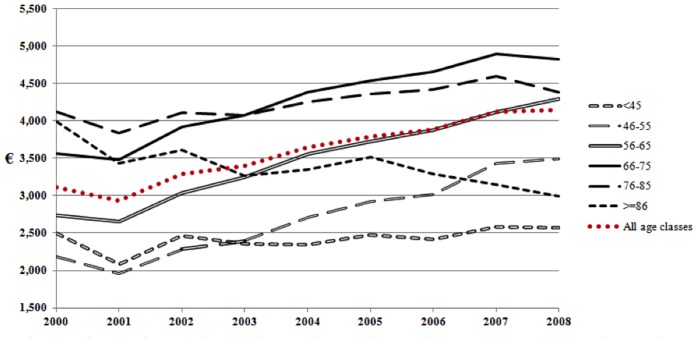
Men expenditure (€/patient-year) by age classes.

Figure 2 and 3 show the overall cost trends during the 9 years of follow up, specific for age classes, of women (figure 2) compared with men (figure 3). Trends of women and men were similar during the follow up: namely, patients 45 years old or younger had a quite stable trend, those older than 85 years showed a decreasing trend, while increasing trends were found in all the other age classes. However, every curve showed lower mean costs among women compared with men, who showed also greater increase with age.

Hospitalizations were the cost driver, overall contributing to 54.2% of total costs. Drugs were the second most important costs (31.5%), while outpatient claims contributed to 14.3% of total costs. As regards hospital costs, 35.6% were attributable to hospital admissions for cardio or cerebro-vascular causes as a main diagnosis, 6% to other possible complications of diabetes, and 4.3% attributable to diabetes as a main diagnosis.

As regards pharmaceutical claims, 33.5% of costs were attributable to Cardiovascular drugs (ATC Class C), 21.8% to class ATC Class A drugs (including anti-diabetic agents), 4.3% of costs were spent for class ATC Class B drugs (2.4% for class B01 drugs). As regards class A drugs, 396 million euro (76.5% of total costs for class A drugs) were attributable to blood glucose lowering drugs (A10), of which 58.9% spent for insulin and 41.1% for non insulin drugs.

**Figure 4:**
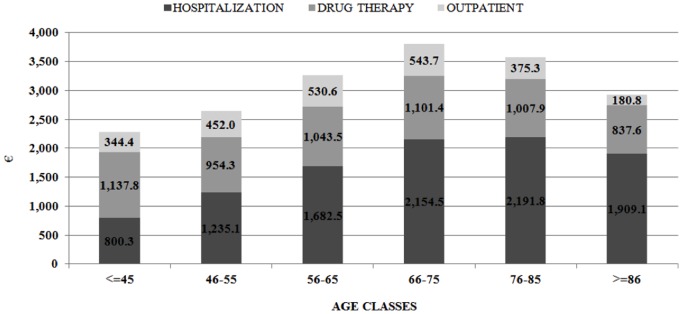
Distribution of overall costs (€/patient-year) by age classes, according to the three main cost categories: hospitalization, drug therapy and outpatient.

Some different contributions of the three classes of costs were found in the different age ranges (Figure 4): the percentage of hospital costs on the total expenditure increased with age, ranging from 35.1% (800 euro/patient-year) of costs among patients 45 years old or younger, to 65.2% (1,900 euro/patient-year) among those older than 85 years. Accordingly, the relative contribute of the other costs decreased: drugs ranged from 49.8% (1,140 euro/patient-year) among ≤45 years old patients, to 28.6% (840 euro/patient-year) of costs in >85 years old subjects, and outpatient costs ranged from 15.1% (340 euro/patient-year) of total costs among ≤45 years old patients, to 6.2% (180 euro/patient-year) in >85 years old subjects.

## Discussion

To the best of our knowledge, this is the most extensive study conducted on the burden of diabetes, focusing on prevalence, mortality and healthcare costs assessed on a large population of diabetes patients observed for 9 years. The conduction of this study was possible with the use of several administrative databases combined together into the data warehouse DENALI with a probabilistic linkage. In the target population a prevalence of diabetes equals to 3.5%, consistently with data obtained from the Italian National Institute of Statistics [14] according which the prevalence in the North of Italy was around 3.9% until 2005. All-cause mortality rate was 43.3 per 1000 subjects. As expected this result was the same reported by Monesi and colleagues [15] for Lombardy diabetic population in 2001. Monesi and colleagues selected diabetic cohort according drugs criteria different from those used in the present work resulting in a slightly smaller cohort. An average of 3,000 € per patient-year were spent in healthcare costs by the RHS, generally higher among men and older people. More than half of costs were attributable to hospital admissions, mostly due to cerebro and cardiovascular reasons (35% of hospital costs). Drugs generated 35% of total costs, one third of these attributable to the use of drugs for cardiovascular reasons. These results were very similar to those reported by Marchesini and colleagues [11] who performed an analysis based on a population-oriented data base covering around 7-million Italian inhabitants: according to their evaluation the direct cost of diabetes was around 2,600 € of which 50% due to hospital admission, 30% to drug treatment and the remaining accounted for by service use.

In their 6-months study Jonsson and colleagues [16] collected retrospectively data from 8 European countries and total healthcare expenditure for type II diabetes in Italy in 1999 was found to be the highest with 2,991 euros per patient-year: the cost was mainly driven by hospitalizations (60%) and 42% of the overall drug costs was attributed to cardiovascular and lipid-lowering drugs. Considering type II diabetes represents around 90% of the overall known diabetic patients, these results were consistent with ours.

As shown by this study, although the administrative databases were not generated with the objective of assessing the epidemiological or economic impact of diseases, they are efficient instruments also for these purposes, since they can provide in a relatively short time period with accurate information directly from the target populations (not from samples only) observed for relevant follow up [17]–[20].

To analyse data of interest that are available in different databases these were merged together using a probabilistic linkage in the data warehouse DENALI. The main advantage of the probabilistic record linkage is that it uses the available individuals’ information to establish a link between database records belonging to the same subject and it does not require identifiers to match exactly. In addition, this method provides the most accurate technique of matching files when they do not share a single common identifier or when there are errors or omission in the identifiers [20]–[21], [23]–[24]. In studies using deterministic record linkage to merge administrative datasets it has been found a 3 to 8% of incorrect patient identification [12], while with the probabilistic record linkage it is possible to identify accurately more than 99% of the study populations.

The present study has some potential limitations. First, individuals with no diabetic exemption, treated with diet only and not hospitalized during the year 2000 could not be included in the study population. However, we do not expect that a consistent part of people with diagnosis of diabetes mellitus was not involved in any of the selection criteria in one year. Furthermore, it was not possible to distinguish between type I and type II diabetes, because neither the exemption code, nor the other selection criteria are specific for type of diabetes. The introduction, in the source of data, of a distinction code between the two types of DM, should allow overcoming this limit for the future research. Third, we probably underestimated healthcare costs, due to the absence of the following information in the administrative databases: costs for disposable such as needles, syringes, glucose test strips and devices like pump and continuing glucose monitors, since they are distributed by the local healthcare agencies; costs for integrated home care and admission in residence for assisted health care, which data are not available through the administrative databases. We did not consider costs of visits to general practitioners’ (GP): almost all residents in Italy are registered at a GP, from whom they receive primary care in case of need. GPs are paid on a capitation basis, regardless of the actual care provided to their assisted persons, hence it is not possible to quantify the costs attributable to the visits for diabetes reasons. Finally, other indicators of the diabetes burden such as loss of productivity, Disability Adjusted Life Years (DALYs), quality of life impairment and Quality Adjusted Life Years (QALYs), were not estimated because the related data were not available in the administrative databases: absenteeism, or impossibility to perform usual activities, to calculate loss of productivity, causes of death for DALYs calculation, and quality of life reported by the target population, expressed as utility index for QALYs calculation. Currently, from data published by the World Health Organization, we are able to indirectly estimate that in Italy, around 552,000 DALYs were attributable to diabetes in the year 2010 [22]. As a future objective it could be useful integrating the administrative databases with new ones in which other types of data, i.e., cause of death, loss of productivity and quality of life elicited with instruments and methods suitable to estimate utilities, are collected and registered, so to generate more complete sources of data for the assessment of burden of diseases.

Nevertheless, this study has been straightforward for its ability to give, in a relatively short time, a quite complete estimate of the burden of DM, promptly providing with information suitable to policy makers for health planning. Although still deserving improvement, the approach used shows to be a fundamental source of readily available and relatively low expensive large amounts of good quality data on general population [23]–[24], for the continuous monitoring of epidemiology and costs, which can be applied also in other disease areas of interest for the decision making process in healthcare.
